# Correction: Vitamin D Supplementation Enhances the Fixation of Titanium Implants in Chronic Kidney Disease Mice

**DOI:** 10.1371/journal.pone.0099972

**Published:** 2014-06-12

**Authors:** The PLOS ONE Staff

## Notice of Republication

This article was republished on May 21st, 2014, due to the figures being published as Supporting Information. The publisher apologizes for this error. Please download the PDF again to view the figures in the corrected article. The originally published, uncorrected article and the republished, corrected article are provided here for reference.

## Supporting Information

File S1 Originally published, uncorrected article
(PDF)

File S2 Republished, corrected article
(PDF)
