# Supplementary material for: Correction: Vitamin D Supplementation Enhances the Fixation of Titanium Implants in Chronic Kidney Disease Mice
Source: PLoS One. 2014 Jun 12;9(6):e99972. doi: 10.1371/journal.pone.0099972 (PMC4055780; doi:10.1371/journal.pone.0099972)
Supplement: File S2 — Republished, corrected article (PDF) [file pone.0099972.s002.pdf]

Vitamin D Supplementation Enhances the Fixation of Titanium Implants in Chronic Kidney Disease Mice

Weiqing Liu[1], Shiwen Zhang[1], Dan Zhao[1], Huawei Zou[1], Ningyuan Sun[1], Xing Liang[1], Michel Dard[2], Beate Lanske[3], Quan Yuan[1]*

1 State Key Laboratory of Oral Diseases, West China Hospital of Stomatology, Sichuan University, Chengdu, China, **2** Department of Periodontology and Implant Dentistry, New York University College of Dentistry, New York, United States of America, **3** Department of Oral Medicine, Infection, and Immunity, Harvard School of Dental medicine, Boston, Massachusetts, United States of America

Abstract

Vitamin D (Vit D) deficiency is a common condition in chronic kidney disease (CKD) patients that negatively affects bone regeneration and fracture healing. Previous study has shown that timely healing of titanium implants is impaired in CKD. This study aimed to investigate the effect of Vit D supplementation on implant osseointegration in CKD mice. Uremia was induced by 5/6 nephrectomy in C57BL mice. Eight weeks after the second renal surgery, animals were given 1,25$(OH)_2D_3$ three times a week intraperitoneally for four weeks. Experimental titanium implants were inserted into the distal end of femurs two weeks later. Serum measurements confirmed decreased 1,25$(OH)_2$D levels in CKD mice, which could be successfully corrected by Vit D injections. Moreover, the hyperparathyroidism observed in CKD mice was also corrected. X-ray examination and histological sections showed successful osseointegration in these mice. Histomorphometrical analysis revealed that the bone-implant contact (BIC) ratio and bone volume (BV/TV) around the implant were significantly increased in the Vit D-supplementation group. In addition, resistance of the implant, as measured by a push-in method, was significantly improved compared to that in the vehicle group. These results demonstrate that Vit D supplementation is an effective approach to improve the fixation of titanium implants in CKD.

Citation: Liu W, Zhang S, Zhao D, Zou H, Sun N, et al. (2014) Vitamin D Supplementation Enhances the Fixation of Titanium Implants in Chronic Kidney Disease Mice. PLoS ONE 9(4): e95689. doi:10.1371/journal.pone.0095689

Editor: Emmanuel A. Burdmann, University of Sao Paulo Medical School, Brazil

Received October 31, 2013; **Accepted** March 30, 2014; **Published** April 21, 2014

Copyright: © 2014 Liu et al. This is an open-access article distributed under the terms of the Creative Commons Attribution License, which permits unrestricted use, distribution, and reproduction in any medium, provided the original author and source are credited.

Funding: This work was supported by grants from the National Natural Science Foundation of China (NSFC 81371173), New Century Excellent Talents in University (NCET-12-0379) and International Team of Implantology (ITI 717_2010) to Q.Y., and the National Institute of Diabetes and Digestive and Kidney Diseases (NIDDK, R01-073944) to B.L. The funders had no role in study design, data collection and analysis, decision to publish, or preparation of the manuscript.

Competing Interests: The authors have declared that no competing interests exist.

* E-mail: yuanquan@scu.edu.cn

Introduction

Chronic kidney disease (CKD) is recognized as a global public health threat. It is a highly prevalent disease with severe complications, such as cardiovascular disease and chronic kidney disease-mineral and bone disorders (CKD-MBD) [1–3]. Screening conducted by nephrologists from all over the world indicates that the incidence of CKD is increasing with ranges of 10.2% to 20% of the population [4–7]. According to a recent cross-sectional survey in China, the prevalence of CKD adults was 10.8%, which means that approximately 119.5 million people are affected by CKD in this rapidly developing country [8].

Declining renal function negatively affects the status of oral health. A recent systematic review showed that poor oral health is a common and often severe side-effect for adults with CKD [9]. Approximately 90% of CKD patients suffer from oral symptoms,including both hard and soft tissues [10–14]. Studies have demonstrated that CKD profoundly influences bone remolding [15] and the structure of the mandible [16]. Furthermore, cross-sectional radiographic examinations indicate that CKD patients experience much more severe alveolar bone loss [17].

Although dental implants have been widely used in clinical settings, opinions regarding implants for CKD patients vary. Both the oral and nephrological literature suggests that dental implant surgery may be contraindicated in patients with significant renal osteodystrophy [18,19]. However, other investigations into the quantity and quality of the alveolar bone of dialysis patients showed that the residual bone volumes were adequate for implant insertion, suggesting this type of treatment is applicable to CKD patients [20]. Previously, we studied the effect of CKD on osseointegration of titanium implants using a uremic mouse model. Although all implants were able to be successfully integrated, CKD significantly decreased the bone-implant strength at an early stage in healing [21]. Therefore, enhancement of dental implant osseointegration in CKD patients remains a challenge.

Vitamin D deficiency is common in CKD patients [22–26] and this deficiency may negatively affect bone regeneration and fracture healing due to its importance in bone metabolism [27]. Low serum 1,25-dihydroxyvitamin D triggers higher levels of serum PTH [28,29], which in turn promotes high bone turnover, exacerbates osteopenia and finally results in cortical bone loss and pathogenesis of osteoporosis [30–32]. Kelly *et al.* [15] showed that vitamin D insufficiency impairs the osseointegration of Ti6Al4V implants in male Sprague-Dawley rats. Alvim-Pereira F *et al.* [33] observed no association between a vitamin D receptor polymorphism (rs731236, TaqI) and dental implant loss. On the other hand, vitamin D supplementation has demonstrated a significant survival advantage in CKD patients [25], and accelerated cellular

events in the process of fracture healing [34]. These findings suggest a potentially beneficial role for vitamin D in promoting implant osseointegration in CKD patients. In this study, we established a CKD mouse model to investigate the effect of vitamin D supplementation on fixation of titanium implants. The results are expected to be a significant contribution to dental implant therapy in CKD patients.

Materials and Methods

Ethics Statement

This study was carried out in strict accordance with the recommendations contained in the Guide for the Care and Use of Laboratory Animals of the National Institutes of Health and the ARRIVE guidelines (http://www.nc3rs.org/ARRIVE). All of the experiments performed were approved by the Subcommittee on Research and Animal Care (SRAC), which serves as the Institutional Animal Care and Use Committee (IACUC) at the Harvard Medical School (protocol number: 03901). All surgery was performed under anesthesia by intraperitoneal injection of a combination of ketamine (100 mg/ml) and xylazine (10 mg/ml), in addition, buprenorphine (0.05 mg/kg) was given for perioperative analgesia to minimize suffering and pain.

Animals

Thirty 9-week-old female C57BL mice (body weight: 22.0+ 2.0 g) were obtained from Charles River Laboratories International Inc. (Wilmington, MA) and randomly assigned to three groups: control(n = 10), CKD(n = 10), and CKD+Vit D(n = 10). The animals were kept under climate-controlled conditions (25°C; 55% humidity; 12 hours of light alternating with 12 hours of darkness) and fed with a standard diet. The workflow is illustrated in Figure 1.

Surgical Procedure to Induce Uremia

CKD is induced in mice by a two-step 5/6 nephrectomy to produce uremia as described previously [21]. Briefly, the first procedure involves electrocautery of the left kidney except for a 2-mm area around the hilum. After 1 week, a total nephrectomy of the right kidney is performed by ligation of the renal hilum and surgical excision. Sham surgery consists of anesthetic administration, flank incision exposing the kidney, and closure of the abdominal wall.

Supplement of Active Vitamin D

The active form of vitamin D [1,25$(OH)_2D_3$], was obtained from Enzo Life Sciences Inc. (Farmingdale, NY) and diluted in saline. Eight weeks after the second kidney surgery, mice in the Vit D-treated group were injected with Vit D (100 ng/kg body weight) 3 times a week until they were sacrificed. Saline was used as a vehicle. The workflow is shown in Fig. S1.

Implant Surgery

Ten weeks after the second surgery (renal ablation), the mice were subjected to implant placement using the method described by Xu *et al.* [35]. Titanium implants with SLA surface (1 mm in diameter and 4 mm in length) were obtained from Institut Straumann AG (Basel, Switzerland). They were cut to the length of 2 mm before insertion. After careful exposure of the distal aspects of the femurs via skin incision and muscle dissection, implant sites were prepared on both sides of the anterior-distal surfaces of the femurs by sequential drilling under cooled sterile saline irrigation with 0.7- and 1.0-mm surgical stainless steel twist drills. Then, the implants were press-fitted into the holes to reach primary stability. After the insertion of the implant, the muscles were carefully sutured with 6-0 silk, which covered the implant and further guaranteed its protection in the biological environment. Then the skin was closed with 5-0 silk.

Serum Biochemical Assays

Two weeks after the insertion of titanium implants, the mice were euthanized by carbon dioxide inhalation. Prior to euthanasia, blood was collected by cheek pouch puncture. Serum biochemistry was performed using commercially available kits: blood urea nitrogen (BUN) (Roche Diagnostics, Indianapolis, IN); 1,25$(OH)_2D$ (Immunodiagnostic Systems Ltd., Fountain Hills, AZ); Calcium and Phosphate (Stanbio Laboratory, Boerne, TX). For the assay of serum ALP activity, the serum was diluted 25 times and measured using a SensoLyte *p*NPP Alkaline Phosphatase Assay Kit (AnaSpec Inc., Fremont, CA).

X-ray Examination and Histological Preparation

Two weeks after implant placement, one femur carrying an implant was harvested from each mouse and fixed in 10% buffered formalin for 1 week at 4°C. Specimens were exposed to X-ray (20 kV, 5 seconds), and then dehydrated and embedded in light-curing epoxy resin (TechnoVit 7200VLC, Hereaus Kulzer, Wehrheim, Germany). Embedded specimens were cut perpendicular to the longitudinal axis of the implants at a site 0.5 mm from its apical end. The specimens were then ground to a thickness of about 50 µm with a grinding system (Exakt Apparatebau, Norderstedt, Germany). Sections were stained with Stevenel's blue and Van Gieson's picro fuchsin stain, and observed by light microscopy.

Histomorphometric Measurement

Images of the implant and peri-implant bone tissues were digitized and histomorphometrically analyzed with NIH Image J (National Institutes of Health, USA). Bone-implant contact (BIC) was calculated as the linear percentage of direct bone-to-implant contact to the total surface of the implant. And the bone volume (BV/TV) in the circumferential zone within 100 µm of the implant surface was calculated. The following formulas were used for analysis.

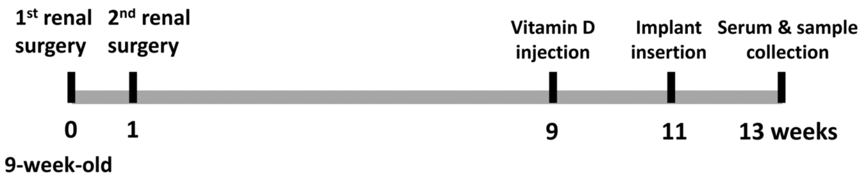

Figure 1. Illustration of the workflow. 1st renal surgery: electrocautery of the left kidney; 2nd renal surgery: a total nephrectomy of the right kidney; Vitamin D injections started at 9th week (100ng/kg body weight, 3 times a week for 4 weeks).
doi:10.1371/journal.pone.0095689.g001

$$Bone-implant\ contact\ (\%) = (sum\ of\ the\ length\ of\ bone\ /\ implant\ contact)/(circumference\ of\ the\ implant) \times 100\%$$

$$Bone\ volume = (bone\ area\ in\ 100\mu m\ zone)/(area\ of\ 100\mu m\ zone) \times 100\%.$$

Implant Biomechanical Push-in Test

The remaining femur from each mouse was harvested and embedded into auto-polymerizing resin with the top surface of the implant level. A test machine (AG-TA electronic universal testing machine, SHIMADZU, Japan) equipped with a pushing rod (diameter = 0.8 mm) was used to load the implant vertically downward at a crosshead speed of 1 mm/min. The push-in value was determined by measuring the peak of the load-displacement curve.

Statistical Analysis

All values were presented as mean ± SD. Statistically significant differences were assessed by ANOVA followed by Tukey's test for multiple comparison, or by independent student t test for comparison between two groups. A p value of less than 0.05 was considered to be statistically significant.

Results

Animals

As anticipated, all mice tolerated the surgical procedures well and survived the full experimental period. No inflammation or infection at the implant site was observed.

Serum Biochemistry

A significant increase in serum BUN was observed in the CKD (53.87±6.32) and CKD+Vit D (58.45±4.33) groups as compared to that of controls (20.12±3.46) ($p<0.05$), indicating the successful establishment of the uremic mouse model (Figure 2A). Vit D injections did not cause a significant change in the serum BUN level. As shown in Figure 2B, Vit D was significantly decreased in the CKD group (27.51±8.35) ($p<0.05$), while supplementing with Vit D (62.44±26.88) restored it to levels comparable to those in control mice (52.35±16.33). Serum PTH in the CKD group was 3.6-fold that observed in the control group ($p<0.05$). It decreased to the levels comparable to the control upon Vit D treatment (Figure 2C). There was no significant difference in serum calcium (Figure 2D) and phosphate (Figure 2E) levels among the three groups. Serum ALP decreased significantly in the Vit D-treated group as compared to the CKD group ($p<0.05$), although it was still significantly higher than that of control (Figure 2F).

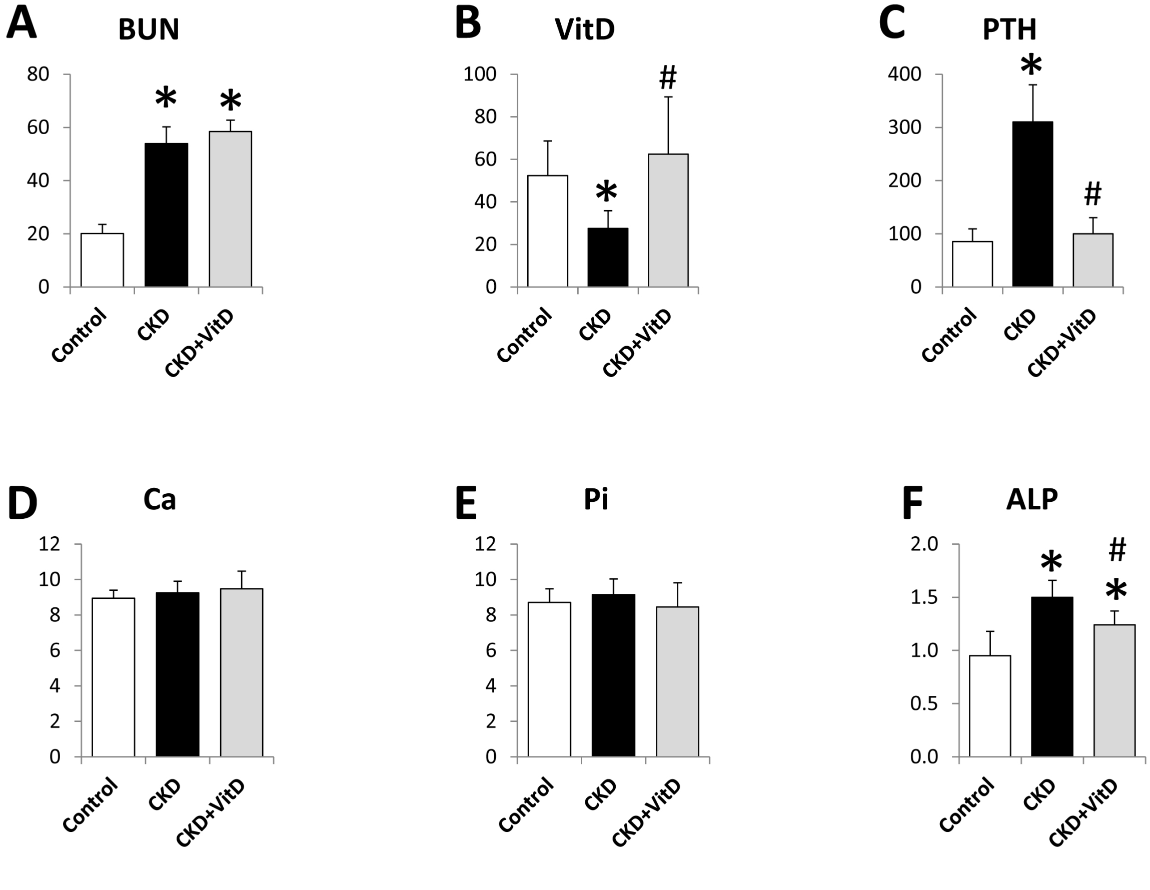

Figure 2. Serum biochemical measurements. (A) Serum BUN; (B) Serum Vitamin D; (C) Serum PTH; (D) Serum calcium; (E) Serum phosphate, and (F) Serum ALP activity. *: $p<0.05$ *vs* Control; #: $p<0.05$ *vs* CKD.
doi:10.1371/journal.pone.0095689.g002

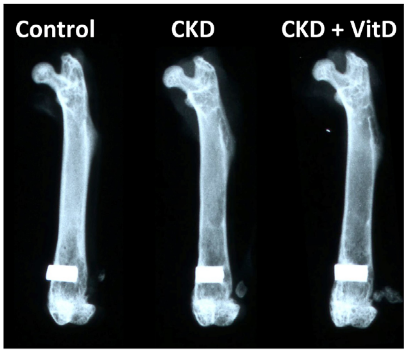

Figure 3. X-ray examination of the femurs after 2-weeks healing. The implants were surrounded with bone without any notable radiotranslucent gap.
doi:10.1371/journal.pone.0095689.g003

X-ray Examination

The *ex vivo* X-ray examination of the femurs showed that the implants were surrounded with bone without any notable radiotranslucent gap, indicating successful osseointegration in all groups (Figure 3).

Histology and Histomorphometry

Examination of histological sections confirmed a direct bone-implant contact in all groups (Figure 4A). Consistent with our previous report [21], the CKD group showed a decrease of bone-implant contact ratio (BIC) when compared to that of control. The contact ratio was successfully restored to the normal level after Vit D treatment. As shown in Figure 4B, the BIC of CKD+VitD group was 75.23±9.92, over 20% higher than that of CKD mice (61.86±10.11) (p<0.05). We then calculated the bone volume (BV/TV) in the circumferential zone within 100 μm of the implant surface and were able to observe a 35% increase after Vit D treatment (Figure 4C).

The Resistance of Implant

In the early healing stage (week 2), the resistance of the implant was significantly higher for the Vit D treated group (15.21±4.11) compared to that of the untreated CKD mice (9.86±1.89), indicating an improved strength of bone–implant integration (Figure 5). No significant difference in integration was observed between the control and CKD+VitD groups.

Discussion

Vitamin D is a pleiotropic hormone that plays a critical role in regulating mineral ion metabolism [28]. It is initially biologically inactive and requires sequential hydroxylations in the liver and kidney to produce its active form, 1,25-dihydroxyvitamin D [28].

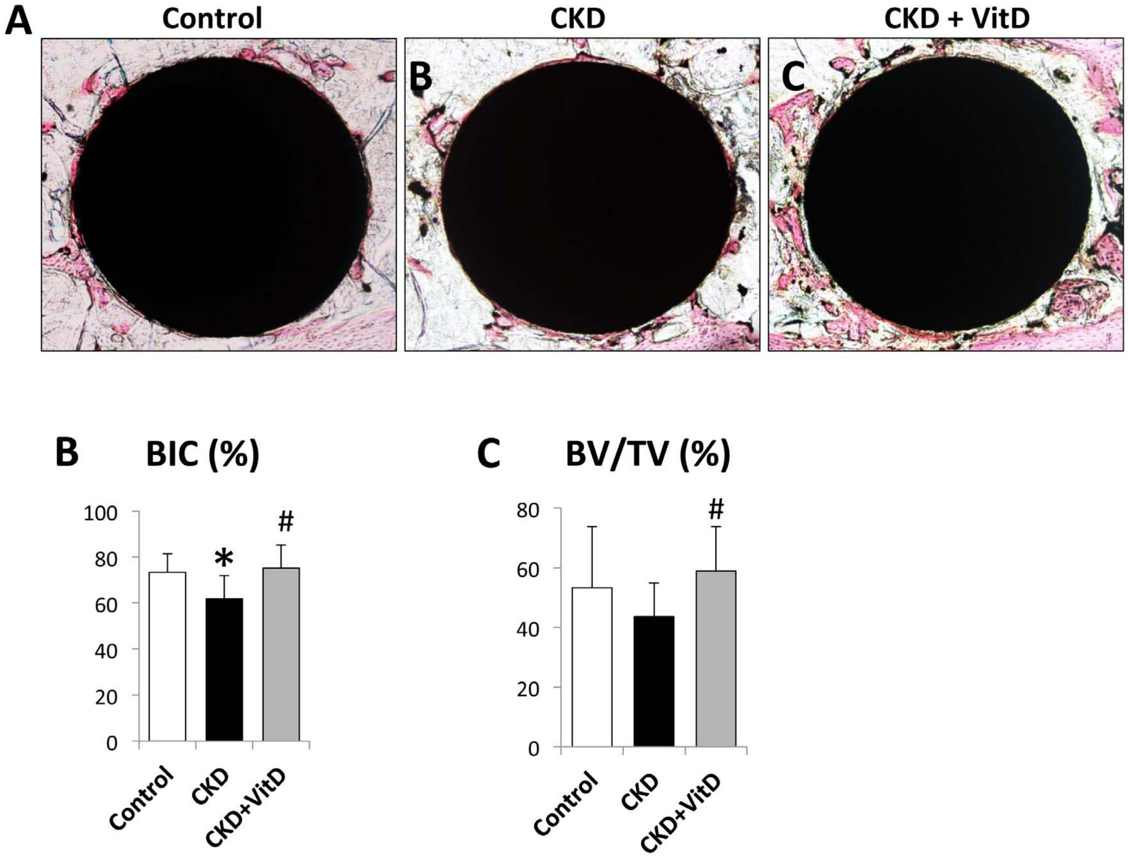

Figure 4. Histological and histomorphometrical analysis of the implants after 2-week healing. (A) Representative images of undecalified sections from all three groups; VitD injection significantly increased (B) Bone-implant contact ratio (BIC, %), and (C) bone volume (BV/TV) in the circumferential zone within 100 μm of the implant surface. *: p<0.05 *vs* Control; #: p<0.05 *vs* CKD.
doi:10.1371/journal.pone.0095689.g004

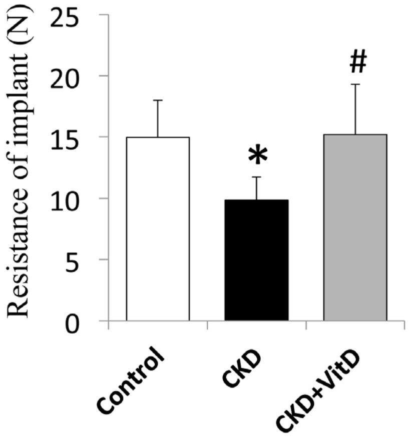

Figure 5. Resistance of push-in tests 2 weeks after implant placement. Vit D supplementation corrected the resistance of implant in CKD. *: $p<0.05$ *vs* control; #: $p<0.05$ *vs* CKD.
doi:10.1371/journal.pone.0095689.g005

Vit D insufficiency is a common medical condition in CKD patients [24], where declining renal function impairs the activity of 1-α hydroxylase (1α-OHase) in kidney, which in turn reduces its ability to convert $25(OH)_2D$ to its activated form, $1,25(OH)_2D$. In this study, we established a uremic mouse model with significantly reduced levels of $1,25(OH)_2D$ using the 5/6 nephrectomy method. These data are consistent with previous studies and similar to the clinical situation in CKD patients [36,37].

Vitamin D deficiency negatively affects bone regeneration, including fracture healing, due to its importance in bone metabolism [27]. Moreover, Kelly *et al.* [15] showed that vitamin D insufficiency decreased the bone-implant contact ratio and the resistance in push-in tests. Recently, Choukroun *et al.* [38] found that Vit D deficiency slows down the process of osseointegration, and also promotes infection in the graft. A genome-wide screening also suggested that Vit D deficiency affects the osseointegration of implants by regulating the circadian rhythm system and the cartilage extracellular matrix [39].

On the other hand, vitamin D supplementation has demonstrated a significant survival advantage in CKD patients [25], including accelerated cellular events in the process of fracture healing [34]. Vitamin D supplementation has been recommended as the primary therapy by the National Kidney Foundation to control secondary hyperparathyroidism and prevent skeletal complications in end stage renal failure patients [40,41]. Two recent studies demonstrated that Vit D supplementation was able to enhance implant fixation in diabetic mellitus rats with Vit D insufficiency [42,43]. Similar results were reported in studies using normal [44] or ovariectomized animals [45,46]. In this study, we successfully corrected the decreased $1,25(OH)_2D$ levels in CKD mice by Vit D injection, and found that both the bone-implant contact (BIC) ratio and the resistance of implant were significantly increased after a 2-week healing period, indicating that Vit D supplementation is an effective approach to improve fixation of titanium implants in CKD.

We also found that Vit D supplementation successfully corrected the increased levels of PTH, a key hormone for bone remodeling. In CKD patients, low serum 1,25-dihydroxyvitamin D triggers higher secretion of PTH [28,29], which precipitates high bone turnover and exacerbates osteopenia and finally results in bone loss [30–32]. A meta-analysis performed by Kandula *et al.* [47] showed that any form of vitamin D (including calcitriol or a vitamin D analog) lowers PTH levels in CKD patients. In this context, we hypothesize that in addition to the direct impact of Vit D on bone healing, the normalization of PTH levels might also contribute to the enhancement of implant fixation. However the direct effect of PTH on osseointegration in CKD needs to be studied further.

It should be noted that this study was performed using a mouse model with the implants placed in the distal end of femurs. Although the rodent model has been widely used for research in implant dentistry[42,45,46], the development and characterization of the femur is different from the jaw bone. A canine or mini pig model might be a better alternative as the implants can be inserted in the jaw bone instead of the femur. However, the establishment of CKD in these large animals is not yet well-established. Clinical trials are expected to confirm whether supplement of Vit D could be a good candidate for clinical approaches to enhance the fixation of titanium implants in CKD patients.

Conclusion

In this study, we found that Vit D supplementation successfully restored serum $1,25(OH)_2D$ levels and corrected the hyperparathyroidism in CKD mice. The bone-implant contact ratio, bone volume around implant and the resistance of the implant were improved at 2 weeks after implantation, indicating that Vit D supplementation is an effective approach to improve the fixation of titanium implants in CKD.

Acknowledgments

We thank Dr. Hans-Peter Weber, Department of Prosthodontics and Operative Dentistry, Tufts University School of Dental Medicine, for the discussion and advice for this study, and Michael Densmore, Department of Oral Medicine, Infection, and Immunity, Harvard School of Dental medicine, for assistance in editing the manuscript.

Author Contributions

Conceived and designed the experiments: QY BL MD. Performed the experiments: WL SZ DZ HZ NS. Analyzed the data: WL XL QY. Contributed reagents/materials/analysis tools: MD BL. Wrote the paper: WL BL QY.

References

1. Moe S, Drueke T, Cunningham J, Goodman W, Martin K, et al. (2006) Definition, evaluation, and classification of renal osteodystrophy: a position statement from Kidney Disease: Improving Global Outcomes (KDIGO). Kidney Int 69: 1945–1953.
2. National Kidney F (2003) K/DOQI clinical practice guidelines for bone metabolism and disease in chronic kidney disease. American journal of kidney diseases : the official journal of the National Kidney Foundation 42: S1–201.
3. Levey AS, Atkins R, Coresh J, Cohen EP, Collins AJ, et al. (2007) Chronic kidney disease as a global public health problem: approaches and initiatives - a position statement from Kidney Disease Improving Global Outcomes. Kidney Int 72: 247–259.
4. Saran R, Hedgeman E, Huseini M, Stack A, Shahinian V (2010) Surveillance of chronic kidney disease around the world: tracking and reining in a global problem. Adv Chronic Kidney Dis 17: 271–281.
5. Kimura G (2007) Predicted prevalence in Japan of chronic kidney disease (CKD). Clinical and experimental nephrology 11: 188–189.
6. Coresh J, Selvin E, Stevens LA, Manzi J, Kusek JW, et al. (2007) Prevalence of chronic kidney disease in the United States. JAMA 298: 2038–2047.
7. Coresh J, Astor BC, Greene T, Eknoyan G, Levey AS (2003) Prevalence of chronic kidney disease and decreased kidney function in the adult US population: Third National Health and Nutrition Examination Survey.

American journal of kidney diseases : the official journal of the National Kidney Foundation 41: 1–12.
8. Zhang L, Wang F, Wang L, Wang W, Liu B, et al. (2012) Prevalence of chronic kidney disease in China: a cross-sectional survey. Lancet 379: 815–822.
9. Ruospo M, Palmer SC, Craig JC, Gentile G, Johnson DW, et al. (2013) Prevalence and severity of oral disease in adults with chronic kidney disease: a systematic review of observational studies. Nephrol Dial Transplant.
10. Jover Cervero A, Bagan JV, Jimenez Soriano Y, Poveda Roda R (2008) Dental management in renal failure: patients on dialysis. Medicina oral, patologia oral y cirugia bucal 13: E419–426.
11. De Rossi SS, Glick M (1996) Dental considerations for the patient with renal disease receiving hemodialysis. Journal of the American Dental Association 127: 211–219.
12. Davidovich E, Schwarz Z, Davidovitch M, Eidelman E, Bimstein E (2005) Oral findings and periodontal status in children, adolescents and young adults suffering from renal failure. Journal of clinical periodontology 32: 1076–1082.
13. Bayraktar G, Kurtulus I, Duraduryan A, Cintan S, Kazancioglu R, et al. (2007) Dental and periodontal findings in hemodialysis patients. Oral Dis 13: 393–397.
14. Davidovich E, Davidovits M, Eidelman E, Schwarz Z, Bimstein E (2005) Pathophysiology, therapy, and oral implications of renal failure in children and adolescents: an update. Pediatric dentistry 27: 98–106.
15. Kelly J, Lin A, Wang CJ, Park S, Nishimura I (2009) Vitamin D and bone physiology: demonstration of vitamin D deficiency in an implant osseointegration rat model. Journal of prosthodontics : official journal of the American College of Prosthodontists 18: 473–478.
16. Lee MM, Chu EY, El-Abbadi MM, Foster BL, Tompkins KA, et al. (2010) Characterization of mandibular bone in a mouse model of chronic kidney disease. Journal of periodontology 81: 300–309.
17. Messier MD, Emde K, Stern L, Radhakrishnan J, Vernocchi L, et al. (2012) Radiographic periodontal bone loss in chronic kidney disease. J Periodontol 83: 602–611.
18. Craig RG, Kotanko P, Kamer AR, Levin NW (2007) Periodontal diseases–a modifiable source of systemic inflammation for the end-stage renal disease patient on haemodialysis therapy? Nephrol Dial Transplant 22: 312–315.
19. Stellingsma C, Vissink A, Meijer HJ, Kuiper C, Raghoebar GM (2004) Implantology and the severely resorbed edentulous mandible. Crit Rev Oral Biol Med 15: 240–248.
20. Dijakiewicz M, Wojtowicz A, Dijakiewicz J, Szycik V, Rutkowski P, et al. (2007) Is implanto-prosthodontic treatment available for haemodialysis patients? Nephrology, dialysis, transplantation : official publication of the European Dialysis and Transplant Association - European Renal Association 22: 2722–2724.
21. Zou H, Zhao X, Sun N, Zhang S, Sato T, et al. (2013) Effect of chronic kidney disease on the healing of titanium implants. Bone 56: 410–415.
22. Ishimura E, Nishizawa Y, Inaba M, Matsumoto N, Emoto M, et al. (1999) Serum levels of 1,25-dihydroxyvitamin D, 24,25-dihydroxyvitamin D, and 25-hydroxyvitamin D in nondialyzed patients with chronic renal failure. Kidney international 55: 1019–1027.
23. Gonzalez EA, Sachdeva A, Oliver DA, Martin KJ (2004) Vitamin D insufficiency and deficiency in chronic kidney disease. A single center observational study. American journal of nephrology 24: 503–510.
24. Rouached M, El Kadiri Boutchich S, Al Rifai AM, Garabedian M, Fournier A (2008) Prevalence of abnormal serum vitamin D, PTH, calcium, and phosphorus in patients with chronic kidney disease: results of the study to evaluate early kidney disease. Kidney international 74: 389–390.
25. Chandra P, Binongo JN, Ziegler TR, Schlanger LE, Wang W, et al. (2008) Cholecalciferol (vitamin D3) therapy and vitamin D insufficiency in patients with chronic kidney disease: a randomized controlled pilot study. Endocrine practice : official journal of the American College of Endocrinology and the American Association of Clinical Endocrinologists 14: 10–17.
26. LaClair RE, Hellman RN, Karp SL, Kraus M, Ofner S, et al. (2005) Prevalence of calcidiol deficiency in CKD: a cross-sectional study across latitudes in the United States. American journal of kidney diseases : the official journal of the National Kidney Foundation 45: 1026–1033.
27. Brinker MR, O'Connor DP, Monla YT, Earthman TP (2007) Metabolic and endocrine abnormalities in patients with nonunions. Journal of orthopaedic trauma 21: 557–570.
28. Lips P (2006) Vitamin D physiology. Progress in biophysics and molecular biology 92: 4–8.
29. Bergwitz C, Juppner H (2010) Regulation of phosphate homeostasis by PTH, vitamin D, and FGF23. Annual review of medicine 61: 91–104.
30. Andress DL (2008) Adynamic bone in patients with chronic kidney disease. Kidney international 73: 1345–1354.
31. Holick MF (2007) Vitamin D deficiency. The New England journal of medicine 357: 266–281.
32. Nickolas TL, Stein E, Cohen A, Thomas V, Staron RB, et al. (2010) Bone mass and microarchitecture in CKD patients with fracture. Journal of the American Society of Nephrology : JASN 21: 1371–1380.
33. Alvim-Pereira F, Montes CC, Thome G, Olandoski M, Trevilatto PC (2008) Analysis of association of clinical aspects and vitamin D receptor gene polymorphism with dental implant loss. Clinical oral implants research 19: 786–795.
34. Jingushi S, Iwaki A, Higuchi O, Azuma Y, Ohta T, et al. (1998) Serum 1alpha,25-dihydroxyvitamin D3 accumulates into the fracture callus during rat femoral fracture healing. Endocrinology 139: 1467–1473.
35. Xu B, Zhang J, Brewer E, Tu Q, Yu L, et al. (2009) Osterix enhances BMSC-associated osseointegration of implants. J Dent Res 88: 1003–1007.
36. Hasegawa H, Nagano N, Urakawa I, Yamazaki Y, Iijima K, et al. (2010) Direct evidence for a causative role of FGF23 in the abnormal renal phosphate handling and vitamin D metabolism in rats with early-stage chronic kidney disease. Kidney international 78: 975–980.
37. Pillar R, MG GL, Rocha LA, Cuppari L, Carvalho AB, et al. (2013) Severe hypovitaminosis D in chronic kidney disease: association with blood pressure and coronary artery calcification. Hypertension research : official journal of the Japanese Society of Hypertension 36: 428–432.
38. Choukroun J, Khoury G, Khoury F, Russe P, Testori T, et al. (2013) Two Neglected Biologic Risk Factors In Bone Grafting And Implantology : High LDL Cholesterol and Low Serum Vitamin D. J Oral Implantol.
39. Mengatto CM, Mussano F, Honda Y, Colwell CS, Nishimura I (2011) Circadian rhythm and cartilage extracellular matrix genes in osseointegration: a genome-wide screening of implant failure by vitamin D deficiency. PloS one 6: e15848.
40. Eknoyan G, Levin A, Levin N (2003) Bone metabolism and disease in chronic kidney disease. American journal of kidney diseases : the official journal of the National Kidney Foundation 42: 1–201.
41. Andress DL (2005) Vitamin D treatment in chronic kidney disease. Seminars in dialysis 18: 315–321.
42. Akhavan A, Noroozi Z, Shafiei AA, Haghighat A, Jahanshahi GR, et al. (2012) The effect of vitamin D supplementation on bone formation around titanium implants in diabetic rats. Dent Res J (Isfahan) 9: 582–587.
43. Wu YY, Yu T, Yang XY, Li F, Ma L, et al. (2013) Vitamin D3 and insulin combined treatment promotes titanium implant osseointegration in diabetes mellitus rats. Bone 52: 1–8.
44. Cho YJ, Heo SJ, Koak JY, Kim SK, Lee SJ, et al. (2011) Promotion of osseointegration of anodized titanium implants with a 1alpha,25-dihydroxyvitamin D3 submicron particle coating. The International journal of oral & maxillofacial implants 26: 1225–1232.
45. Dvorak G, Fugl A, Watzek G, Tangl S, Pokorny P, et al. (2012) Impact of dietary vitamin D on osseointegration in the ovariectomized rat. Clinical oral implants research 23: 1308–1313.
46. Zhou C, Li Y, Wang X, Shui X, Hu J (2012) 1,25Dihydroxy vitamin D(3) improves titanium implant osseointegration in osteoporotic rats. Oral surgery, oral medicine, oral pathology and oral radiology 114: S174–178.
47. Kandula P, Dobre M, Schold JD, Schreiber MJ Jr, Mehrotra R, et al. (2011) Vitamin D supplementation in chronic kidney disease: a systematic review and meta-analysis of observational studies and randomized controlled trials. Clinical journal of the American Society of Nephrology : CJASN 6: 50–62.
